# Implications of monogenic bicuspid aortic valve (BAV) forms among sporadic BAV patients

**DOI:** 10.1038/s41431-025-01909-7

**Published:** 2025-07-11

**Authors:** Christopher M H Bruenger, Frank Oeffner, Laura L Koebbe, Sebastian Zimmer, Verena Veulemans, Matti Adam, Jessica Bigge, Stefanie Heilmann-Heimbach, Malte Kelm, Stephan Baldus, Markus M Nöthen, Georg Nickenig, Carlo Maj, Baravan Al-Kassou, Johannes Schumacher

**Affiliations:** 1 Institute of Human Genetics, Philipps University of Marburg, Marburg, Germany; 2 Department of Medicine II, Heart Center Bonn, University of Bonn and University Hospital Bonn, Bonn, Germany; 3 Department of Cardiology, Pneumology and Angiology, University Hospital Duesseldorf, Duesseldorf, Germany; 4 Department of Medicine III, Heart Center Cologne, University Hospital Cologne, Cologne, Germany; 5 Institute of Human Genetics, University of Bonn and University Hospital Bonn, Bonn, Germany; 6 Next Generation Sequencing Core Facility, Medical Faculty, University Bonn, Bonn, Germany

**Keywords:** Genetics research, Congenital heart defects

## Abstract

Bicuspid aortic valve (BAV) represents the most common congenital heart defect and is genetically heterogeneous. While the majority of cases results from common risk variants that confer disease cumulatively, a small proportion of BAV cases has a monogenic etiology where penetrant rare variants (RVs) in single genes are disease causing. We assessed the proportion of monogenic BAV cases in 740 non-syndromic and non-familial BAV patients that should be representative for cardiovascular centers of maximum care. We used next generation sequencing- (NGS-) based single-molecule molecular inversion probes (smMIPs) and analyzed all monogenic BAV genes that have been identified so far (*NOTCH1*, *SMAD6*, *ROBO4*, *GATA4*, *GATA6*, and *ADAMTS19*). In these genes, we identified potential damaging RVs in 2% of our patients, which were not significantly enriched compared to 726 population-based controls. We conclude that the contribution of monogenic BAV forms is only small among non-syndromic and sporadic BAV patients.

Non-syndromic bicuspid aortic valve (BAV) is the most common congenital heart defect with an estimated prevalence of 0.5–2% in the population [1]. BAV is of major clinical relevance because of its high lifetime risk of complications such as aortic valve stenosis (AS), aortic valve insufficiency (AI) or thoracic aortic aneurysm (TAA) [1]. The majority of non-syndromic BAV cases have a multifactorial aetiology, where common genetic variants confer disease risk in a cumulative fashion [2]. Only a small proportion of non-syndromic BAV cases have a monogenic aetiology, where penetrant rare variants (RVs) in single genes are disease causing [3–8]. However, due to incomplete penetrance and variable expressivity it can be difficult to identify monogenic BAV cases.

In the present study, we aimed to assess the proportion of monogenic forms among sporadic BAV patients. Figure 1 shows the design and the main results of this study. As patients, we included 740 non-syndromic BAV cases (*N* = 191 females, *N* = 549 males) that were recruited at cardiovascular centres of three University Hospitals in Western Germany (Bonn, Cologne, Duesseldorf) between 2016 and 2020. All patients were of European descent and were referred to the hospitals due to BAV complications (*N* = 399 with AS, *N* = 536 with AI, *N* = 270 with TAA, overlapping phenotypes possible). In all patients BAV diagnosis was confirmed using transthoracic/transoesophageal echocardiography or computed tomography. There was no evidence for syndromic BAV in all patients, i.e. clinical symptoms were excluded that are – among others – characteristic of Turner, Down, Loeys-Dietz or velocardiofacial syndromes. In addition, familial BAV was excluded based on patient self-reports. Although we cannot exclude the presence of syndromic or familial cases entirely, the present sample should be representative for non-syndromic BAV cases at cardiovascular centres of maximum care and served to assess whether there is a significant proportion of monogenic BAV forms among sporadic patients. As controls we used 726 population-based European individuals (*N* = 187 females, *N* = 539 males) that were part of the Heinz Nixdorf Recall Study (HNR), which represents a large investigation of cardiovascular disease among individuals living in or near the city of Essen in Western Germany (https://imibe.uk-essen.de/information-for-scientists/). All controls were free of self-reported cardiovascular disease, underwent baseline questionnaires and testing including screening for subclinical cardiovascular disease, but not particularly for the presence of BAV. Informed written consent was obtained from all patients and ethics committees at all involved University Hospitals approved this study.

**Fig. 1:**
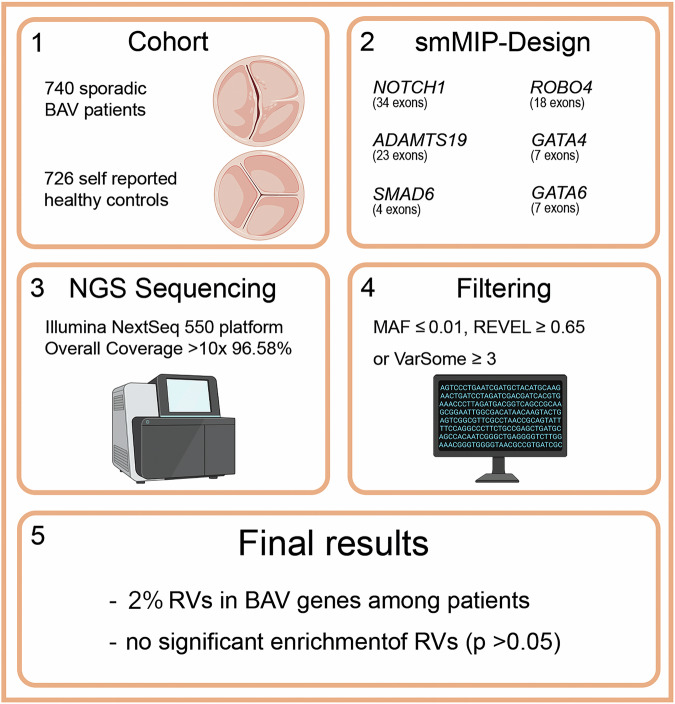
Overview of the study design and findings. The study cohort consisted of confirmed BAV patients and self-reported healthy controls. The population-based control cohort was not screened for BAV and we cannot rule out the presence of BAV in the range of its population-based prevalence (0.5–2% [1]). Variant filtering included a MAF ≤ 0.01 in the gnomAD non-Finish European population. In addition, only variants that were predicted as being deleterious according to REVEL and/or VarSome were used. BAV Bicuspid aortic valve, NGS Next generation sequencing, smMIP single molecule Molecular Inversion probe, MAF Minor allele frequency, REVEL Rare Exome Variant Ensemble Learner scores [11], VarSome VarSome: the human genomic variant search engine [10], RVs Rare variants. Created with images from BioRender.

To assess the impact of monogenic BAV forms in our sample we carried out a targeted sequence approach using next generation sequencing (NGS) and single-molecule molecular inversion probes (smMIPs), which represents a high-throughput screening method for the simultaneous analysis of specific genomic regions [9]. In total, we designed 216 smMIP-primers in order to cover the coding regions of all 6 monogenic genes that have been implicated in non-syndromic BAV to date [3–8]. These are *NOTCH1* (34 exons), *SMAD6* (4 exons), *ROBO4* (18 exons), *GATA4* (7 exons), *GATA6* (7 exons), and *ADAMTS19* (23 exons). Supplementary Table 1 provides information on all primers that were used. After optimizing our smMIP assay [9], we sequenced the final pooled MIP libraries in a paired-end 150 bp run on a NextSeq550 (Illumina, San Diego, CA, USA). After standard quality control (QC) and processing of smMIP sequencing data [9] we identified a total of 1164 genetic variants that were covered by >10 reads. Averaged across all genes, we achieved an overall 10×/20× coverage of 96.58%/80.72% with a mean depth of 1234.01 (*NOTCH1* 96.78%/86.43% mean depth 971.45, *SMAD6* 77.34%/55.19% mean depth 288.93, *ROBO4* 99.46%/98.48% mean depth 1142.10, *GATA4* 97.46%/80.85% mean depth 606.38, *GATA6* 99.23%/80.73% mean depth 708.95, *ADAMTS19* 99.51%/98.24% mean depth 2712.89*)*. Of those we only considered RVs with a minor allele frequency (MAF) ≤ 0.01 and a deleteriousness threshold for genetic variants defined by a VarSome score[10] ≥ 3 or Rare Exome Variant Ensemble Learner (REVEL) scores[11] ≥ 0.65. We used VarSome and REVEL, because both tools showed previously convincing results in predicting potentially deleterious RVs [12, 13].

Application of our filter criteria identified 12 predicted deleterious RVs in 15 patients among the dominant BAV genes (*NOTCH1, SMAD6, ROBO4, GATA4*, and *GATA6*). This refers to 2% of cases (15/740) with *NOTCH1* showing the highest number of RVs. We identified 6 predicted deleterious RVs among 9 patients in *NOTCH1*, which is more than twice the number of RVs carriers we observed in the remaining dominant BAV genes together (Table 1). These were 3 predicted deleterious RVs in *ROBO4*, two in *GATA4* and one in *SMAD6* (Table 1). In *GATA6* we did not identify any RV in patients. However, for none of these genes we found a significant enrichment of RVs compared to controls applying SKAT-O [14] as gene-based burden test (*P* > 0.05, Table 1). In addition, no compound heterozygous or homozygous predicted deleterious RVs were present among patients in the recessive BAV gene *ADAMTS19* (Table 1). Supplementary Table 2 lists all RVs that were found among all participants and genes, including their chromosomal position and types of base and amino acid substitution as well as the VarSome and REVEL scores. In addition, we performed a separate analysis of loss-of-function (LoF) RVs that also showed no enrichment in any of the studied genes among BAV patients (*P* > 0.05, Supplementary Table 3). Furthermore, no enrichment of RVs was observed in our patients using AlphaFold [15] or AlphaMissense [16] as alternative tools for the prediction of deleterious RVs (*P* > 0.05, Supplementary Table 4). We also tested the missense variant rs61751489 (V2285I) in *NOTCH1*, which has been previously reported as BAV-relevant [17], but was not predicted as deleterious by our prediction tools. Also this variant showed no enrichment among our BAV patients compared to controls (*P* > 0.05, Supplementary Table 5). Due to the limited size of our control sample, we further performed a gene-based burden test using non-Finnish Europeans from gnomAD as controls (*N* = 56,885 individuals) (https://gnomad.broadinstitute.org/). Also in this analysis, we found no enrichment of RVs in all 6 genes among our patients compared to gnomAD controls (*P* > 0.05, Supplementary Table 6).

**Table 1:** Predicted deleterious RVs identified in the monogenic BAV genes *NOTCH1*, *SMAD6*, *ROBO4*, *GATA4*, *GATA6*, and *ADAMTS19* among patients and controls (number (No) of carrier).

Gene	No of different variants in cases	No of mutation carrier in patients	No of different variants in controls	No of mutation carrier in controls	No of different variants overall	*P* value (Burden Analysis)	OR (Haldane-Anscombe correction)	95% CI
*NOTCH1*	6	9	3	6	9	0.458	1.97	0.49–7.90
*GATA4*	2	2	1	2	3	0.985	1.965	0.18–21.72
*GATA6*	0	0	1	1	1	0.313	0.327	0.01–8.03
*SMAD6*	1	1	2	3	3	0.552	0.5	0.04–5.41
*ROBO4*	3	3	0	0	3	0.086	6.9	0.36–133.74
*ADAMTS19*	1	3	1	1	2	—	—	—

*P* values, odds ratios (OR) and the corresponding 95% confidence intervals (95% CI) are shown for dominant BAV genes. *ADAMTS19* represents a recessive BAV gene and no compound heterozygous or homozygous RVs that were predicted as deleterious were found among patients.

We next tested whether the 9 patients with predicted deleterious RVs in *NOTCH1* show clinical characteristics that distinguish them from the other BAV cases (Supplementary Table 7). Here, patients with RVs showed no distinct pattern of BAV complications (AS: 5 in RV carrier versus 394 in non-RV carrier (*P* = 0.724), AI: 7 in RV carrier versus 529 in non-RV carrier (*P* = 0.861), TAA: 4 in RV carrier versus 266 in non-RV carrier (*P* = 0.670)). However, we could not test, whether the 9 patients with predicted deleterious RVs in *NOTCH1* required surgery or interventions earlier in life than those without RVs, as corresponding clinical data were not available.

In summary, we found that only 2% of sporadic patients carry predicted deleterious RVs in the monogenic BAV genes *NOTCH1*, *SMAD6*, *ROBO4*, *GATA4*, *GATA6*, and *ADAMTS19*. More than twice as many concerned *NOTCH1*, where RVs were found most frequently. Our findings are in accordance with previous studies where no significant burden of mutations has been found in known BAV-genes among sporadic patients [17–19]. Although we identified monogenic subtypes of BAV only in a small proportion of our sample, we cannot exclude the presence of deleterious RVs in genes that typically lead to syndromic BAV and have not been analysed in our study. Accordingly, it has been shown previously that deleterious RVs in those genes can also been involved in sporadic BAV patients [20]. In addition, the 10x sequencing coverage was below 90% for *SMAD6*. We, thus, cannot rule out that we have missed carriers of deleterious RVs in *SMAD6* among our cases.

## Supplementary information

Supplementary Table 1

Supplementary Table 2

Supplementary Table 3

Supplementary Table 4

Supplementary Table 5

Supplementary Table 6

Supplementary Table 7

## Data Availability

For data protection reasons, the smMIPs data set is only available on reasonable request to the corresponding author.
